# Supplementary material for: Splice-Junction-Based Mapping of Alternative Isoforms in the Human Proteome
Source: Cell Rep. Author manuscript; Available in PMC 2020 Jan 15. (PMC6961840; doi:10.1016/j.celrep.2019.11.026)
Supplement: 3 [file NIHMS1546469-supplement-3.zip › DF2/PXD006675/SmoothMuscleCells-114-Q9UN86-STTPPPAEPVSLPQEPPKPR.pdf]

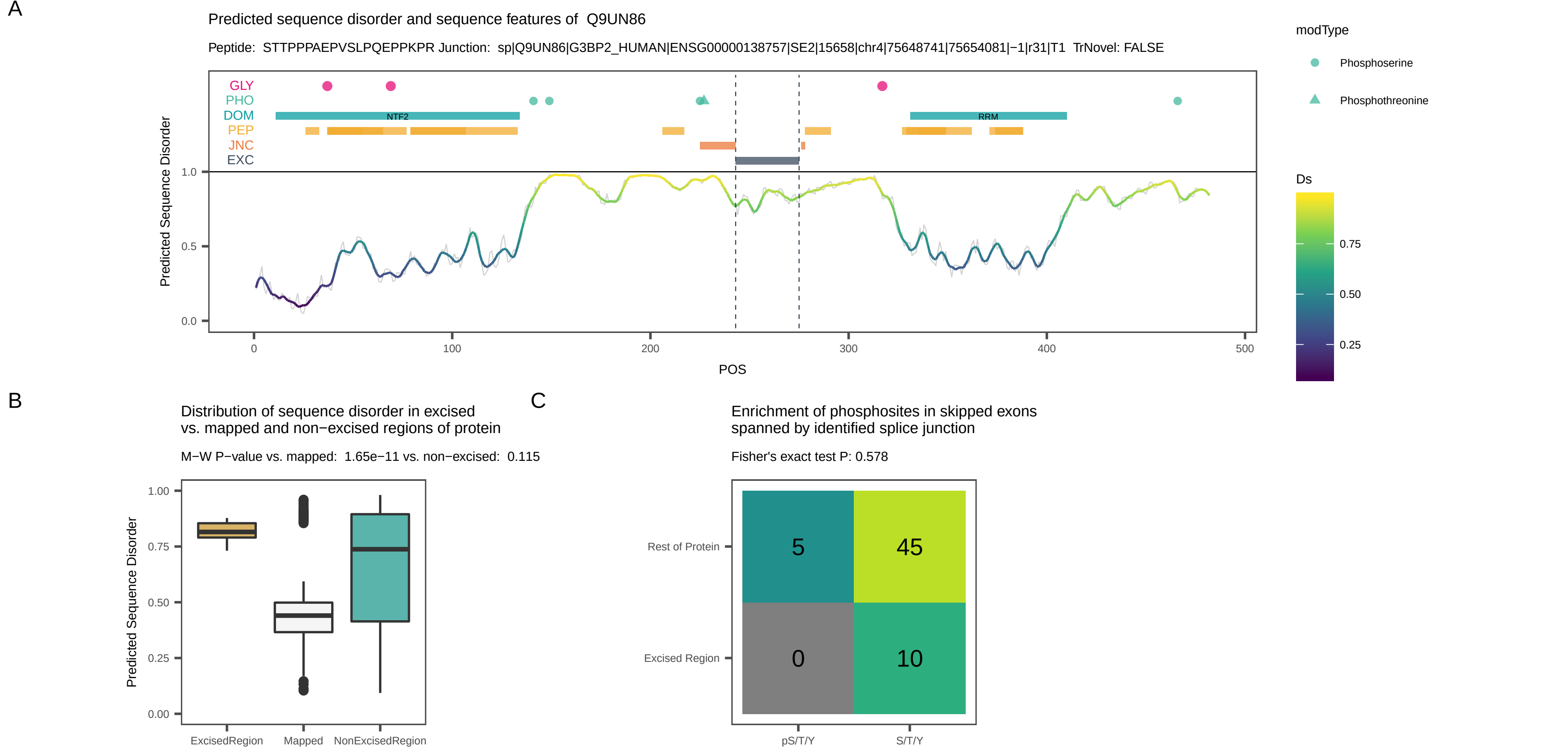

A
Predicted sequence disorder and sequence features of Q9UN86
Peptide: STTPPPAEPVSLPQEPPKPR Junction: sp|Q9UN86|G3BP2_HUMAN|ENSG00000138757|SE2|15658|chr4|75648741|75654081|−1|r31|T1 TrNovel: FALSE
GLY
PHO
DOM
PEP
JNC
EXC
NTF2
RRM
Predicted Sequence Disorder
1.0
0.5
0.0
0
100
200
300
400
500
POS
modType
Phosphoserine
Phosphothreonine
Ds
0.75
0.50
0.25
B
Distribution of sequence disorder in excised vs. mapped and non−excised regions of protein
M−W P−value vs. mapped: 1.65e−11 vs. non−excised: 0.115
Predicted Sequence Disorder
1.00
0.75
0.50
0.25
0.00
ExcisedRegion
Mapped
NonExcisedRegion
C
Enrichment of phosphosites in skipped exons spanned by identified splice junction
Fisher's exact test P: 0.578
Rest of Protein
Excised Region
5
45
0
10
pS/T/Y
S/T/Y
